# Pregnancy complicated with low-grade appendiceal mucinous tumor: A case report

**DOI:** 10.1097/MD.0000000000044354

**Published:** 2025-09-12

**Authors:** Beijiao Wu, Ling Ai

**Affiliations:** a Jiaxing Maternity and Child Health Care Hospital, Jiaxing, Zhejiang Province, China; b Jiaxing Women and Children’s Hospital, Wenzhou Medical University, Jiaxing, Zhejiang Province, China.

**Keywords:** case report, low-grade appendiceal mucinous neoplasm, pregnancy, prenatal ultrasound, pseudomyxoma peritonei, surgical management

## Abstract

**Rationale:**

Low-grade appendiceal mucinous neoplasm (LAMN) is a rare neoplastic entity, especially during pregnancy. Early prenatal detection remains exceedingly rare, and no consensus exists regarding the optimal timing or strategy for surgical intervention during pregnancy.

**Patient concerns:**

A 31-year-old pregnant woman at 27 weeks of gestation presented with mild abdominal pain accompanied by diarrhea. Routine prenatal ultrasonography identified a cystic mass in the right lower quadrant of the abdomen, measuring 76 × 21 × 26 mm, exhibiting the characteristic “onion skin sign.”

**Diagnoses:**

Prenatal ultrasonography raised suspicion of LAMN. Follow-up imaging and definitive histopathological evaluation following surgical resection confirmed the diagnosis of LAMN.

**Interventions:**

Given the patient’s stable condition and lack of symptom progression, the multidisciplinary team elected to pursue expectant management with serial ultrasonography. At 40 weeks of gestation, she underwent an elective cesarean section, followed by an appendectomy and partial ileocecal resection. Surgery was performed with precautions to prevent mucinous dissemination.

**Outcomes:**

Histopathological analysis confirmed LAMN in the absence of rupture or peritoneal dissemination. The patient experienced an uneventful postoperative recovery, and both mother and infant remained in good health throughout 12 months of follow-up, with no radiological or clinical evidence of recurrence.

**Lessons:**

This case highlights the feasibility of accurate prenatal diagnosis of LAMN via ultrasonography and emphasizes the importance of multidisciplinary management in optimizing maternal-fetal outcomes. Expectant management until full-term delivery, followed by coordinated surgical intervention, may represent a safe and effective approach in selected cases of pregnancy complicated by LAMN.

## 1. Introduction

Low-grade appendiceal mucinous neoplasm (LAMN) is a rare entity, accounting for 0.2% to 0.4% of all appendectomy specimens and primarily affecting middle-aged to elderly individual.^[1,2]^ Accumulation and peritoneal dissemination of mucin from the tumor cavity can result in pseudomyxoma peritonei (PMP), a condition that is typically progressive and fatal. Only a few cases of pregnancy complicated by LAMN have been reported. Owing to the wide variability in clinical presentation, LAMN is often diagnosed intraoperatively or postoperatively.

Beyond organ-specific reports, rare tumors diagnosed during pregnancy face distinctive diagnostic and therapeutic constraints, including limited imaging options and complex coordination of delivery and oncologic intervention. A recent synthesis by La Verde et al underscores these challenges and the need for individualized, multidisciplinary strategies across gestational malignancies.^[3]^ Complementing this, Goidescu et al highlight recurrent diagnostic delays, imaging limitations, and delivery–surgery timing trade-offs in pregnancy through a case and literature review.^[4]^ To our knowledge, this is the first reported case of an asymptomatic pregnant woman diagnosed with LAMN through routine prenatal ultrasonography during the second trimester. The tumor was incidentally detected at 27 weeks of gestation, in the absence of specific gastrointestinal symptoms. This rare presentation not only contributes novel insights to the limited literature on LAMN in pregnancy but also underscores the potential role of prenatal imaging in detecting silent yet potentially serious abdominal pathology at an early stage.

## 2. Case report

A 31-year-old pregnant woman underwent frozen embryo transfer on March 8, 2023 (gestational age 3 + 2 weeks), for the treatment of primary infertility. She had a medical history of hepatitis B infection and penicillin allergy. Her prenatal evaluations remained unremarkable until 27 + 0 weeks of gestation.

On August 23, 2023 (27 + 0 weeks of gestation), she presented to the emergency department with 4 episodes of diarrhea and abdominal pain, accompanied by mild lower abdominal discomfort. Physical examination revealed a soft, nontender abdomen. Laboratory tests, including routine bloodwork, C-reactive protein, and serum electrolytes, were all within normal limits. She was diagnosed with late threatened abortion and acute gastroenteritis. Her symptoms improved significantly following oral treatment with montmorillonite powder and bifidobacterium triple viable capsules.

**Figure 1.**
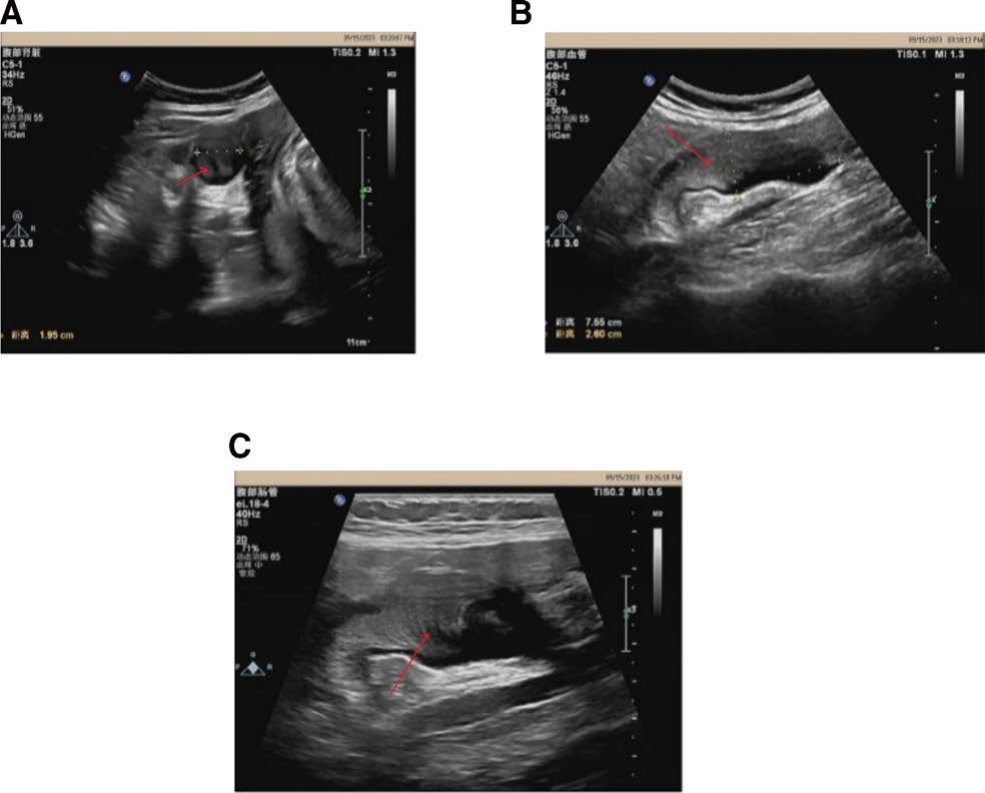
Appendix ultrasound image. (A) Transverse section of the appendix under abdominal ultrasound. (B) Longitudinal section of the appendix under abdominal ultrasound. (C) Typical “onion skin” sign on abdominal ultrasound. All red arrows indicate the typical “onion skin sign.”

At a routine prenatal checkup on September 13, 2023 (30 + 0 weeks of gestation), abdominal ultrasonography revealed a cystic mass measuring 76 × 21 × 26 mm in the right lower quadrant. The mass appeared anechoic with comb-like internal echoes and demonstrated a distinct “onion skin sign” (Fig. 1), suggestive of LAMN. The patient was referred to the general surgery department. As she remained asymptomatic (reporting no abdominal pain, distension, or gastrointestinal symptoms), surgical intervention was deferred. Conservative management was pursued with serial ultrasonographic evaluations at 32 + 0 weeks (September 27, 2023), 34 + 0 weeks (October 11, 2023), 36 + 0 weeks (October 25, 2023), and 38 + 0 weeks (November 8, 2023); no significant increase in the size of the mass or new symptoms were observed during this period.

At 39 + 5 weeks of gestation (November 20, 2023), the patient was admitted for delivery. Routine admission testing was unremarkable, except for positive results for Group B Streptococcus and hepatitis B surface antigen. A multidisciplinary team (MDT) recommended a cesarean section with concurrent appendectomy.

On November 22, 2023 (40 + 0 weeks), a lower-segment cesarean section was performed under combined spinal–epidural anesthesia, resulting in the delivery of a healthy male infant (birth weight: 3320 g; Apgar scores: 9 at 1 and 5 minutes). This was followed by an appendectomy and partial ileocecal resection.

After cesarean closure, we re-prepped with a clean set, changed gloves, placed a wound protector, and isolated the appendix with moist pads. A no-touch, low-suction en-bloc appendectomy with partial ileocecectomy was performed for the densely adherent base without entering the lumen. The specimen was bagged and removed via the protector; the cavity was irrigated/aspirated and systematically surveyed; gloves were re-changed, and no rupture or mucin leakage occurred.

**Figure 2.**
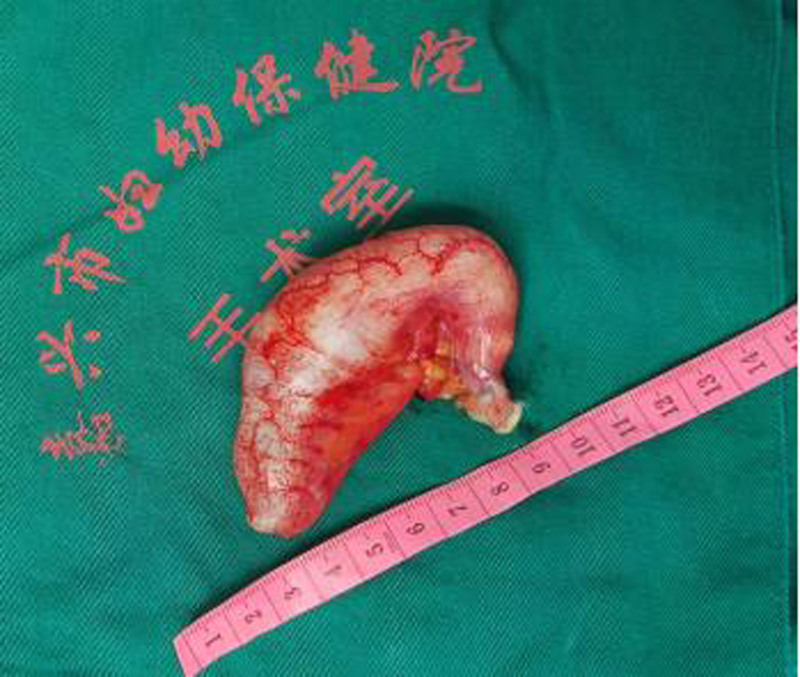
Intraoperative gross case specimen.

**Figure 3.**
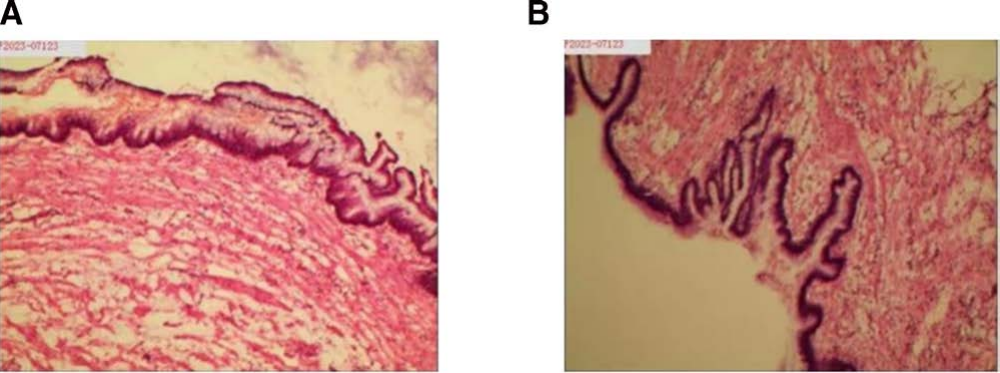
Intraoperative frozen pathology section. (A) Frozen section of a low-grade mucinous neoplasm. (B) Chronic inflammation at the surgical resection margin.

**Figure 4.**
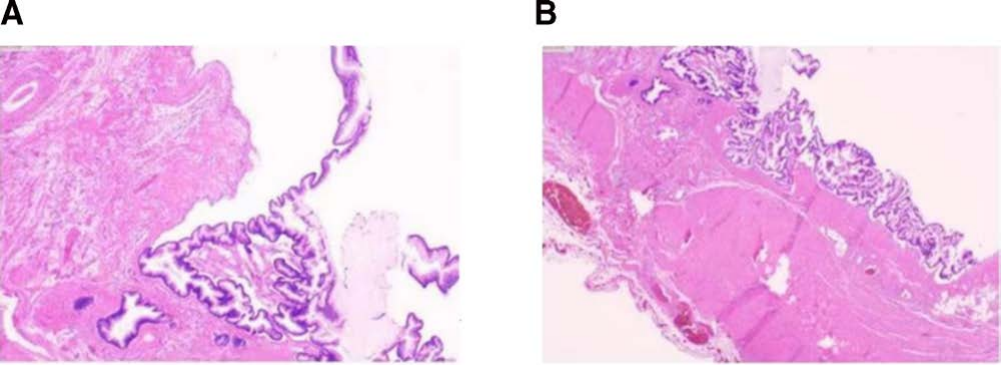
Conventional pathology section. (A) Low-grade appendiceal mucinous neoplasm. (B) LAMN with superficial mucinous epithelium. LAMN = low-grade appendiceal mucinous neoplasm.

Intraoperatively, the appendix appeared enlarged and tense, measuring ~8 × 4 cm, with marked thickening of the body and head, and a base diameter of 0.6 cm (Fig. 2). Due to firm adhesion of the appendix to the ileocecal region, and to ensure complete excision with negative margins, a partial ileocecal resection was also performed. No rupture or mucin leakage was observed, and the adnexal structures appeared normal. Intraoperative frozen section confirmed a low-grade mucinous neoplasm with chronic inflammation at the resection margins (Fig. 3). Final histopathological analysis corroborated the diagnosis (Fig. 4). Immunohistochemical analysis showed cytokeratin 19 (CK19) (+), CK20 (+), CK7 (–), carcinoembryonic antigen (CEA) (+), Ki-67 (~20%), and strong p53 expression (~80%). All mismatch repair proteins, including MLH1, PMS2, MSH2, and MSH6, were retained.

**Figure 5.**
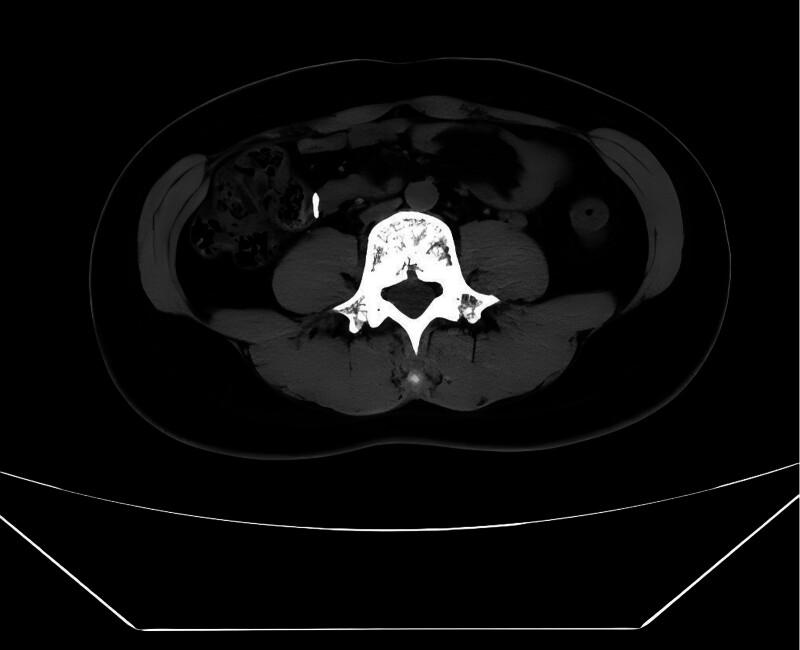
Postoperative abdominal CT images at 6 months (April 2024). CT = computed tomography.

The patient was discharged in good condition on postoperative day 5 (November 27, 2023). A follow-up computed tomography (CT) scan performed at 2 weeks postpartum (December 11, 2023) revealed no abnormalities. At the 6-week postpartum visit (January 8, 2024), both maternal and neonatal evaluations were unremarkable, with no complications observed. Follow-up CT scans at 4 months postpartum/6 months postoperative (April 8, 2024) and 11.5 months postpartum/1 year postoperative (November 15, 2024) showed no evidence of recurrence, and the patient remained asymptomatic (Fig. 5). The complete timeline of the patient’s clinical course is presented in Table 1.

**Table 1:** Timeline of clinical events.

Date	Gestational age/postpartum time	Event
February 15, 2023	0 + 0 weeks	Last menstrual period
March 8, 2023	3 + 2 weeks	Frozen embryo transfer for primary infertility
August 23, 2023	27 + 0 weeks	Presented with abdominal pain and diarrhea; diagnosed with acute gastroenteritis and threatened abortion; labs normal; treated symptomatically
September 13, 2023	30 + 0 weeks	Routine prenatal ultrasound revealed a 76 × 21 × 26 mm cystic mass in right lower abdomen with “onion skin sign”; referred to general surgery
September 27, 2023	32 + 0 weeks	Follow-up ultrasound: mass size stable, no new symptoms
October 11, 2023	34 + 0 weeks	Follow-up ultrasound: mass unchanged
October 25, 2023	36 + 0 weeks	Follow-up ultrasound: mass unchanged
November 8, 2023	38 + 0 weeks	Follow-up ultrasound: mass unchanged
November 20, 2023	39 + 5 weeks	Hospitalized for delivery; GBS positive; MDT planned cesarean section with concurrent appendectomy and partial ileocecal resection
November 22, 2023	40 + 0 weeks	Lower-segment cesarean section performed; healthy male infant delivered (3320 g, Apgar 9/9); appendectomy + partial ileocecal resection performed; intraoperative frozen section confirmed low-grade mucinous neoplasm
November 27, 2023	Postop day 5	Discharged in good condition
December 11, 2023	2 weeks postpartum	Follow-up abdominal CT: no abnormalities
January 8, 2024	6 weeks postpartum	Routine postpartum follow-up: maternal and infant exams normal, no complications
April 8, 2024	4 months postpartum/6 months postop	Abdominal CT follow-up: no recurrence (Fig. 5)
November 15, 2024	11.5 months postpartum/1 year postop	Annual follow-up: patient asymptomatic, no evidence of recurrence

CT = computed tomography, GBS = group B streptococcus, MDT = multidisciplinary team.

## 3. Discussion

The incidence ratio of LAMN between males and females is ~1:4, with reported onset ages ranging from 11 to 90 years, peaking between 61 and 79 years, and a mean age at diagnosis of 55 years.^[5,6]^ If an appendiceal mucinous neoplasm penetrates the appendiceal wall and spreads into the peritoneal cavity, it can give rise to a severe clinical syndrome known as PMP.^[7]^ Clinical manifestations of the disease vary considerably among individuals. The most frequent presentations resemble those of acute or chronic appendicitis. More than 50% of patients present with acute or chronic right lower quadrant pain,^[8]^ while ~25% remain asymptomatic, with the condition often detected incidentally during physical examination or surgery.^[9]^ Other symptoms may include weight loss, nausea, vomiting, and loss of appetite. In the present case, the patient’s abdominal pain and diarrhea significantly improved with symptomatic treatment, which initially led to a presumptive diagnosis of acute gastroenteritis (a condition difficult to distinguish based on clinical symptoms alone). Therefore, preoperative diagnosis remains challenging, with many cases identified intraoperatively or postoperatively.

Preoperative diagnosis of LAMN primarily relies on imaging modalities. On CT, a well-circumscribed, round, low-density, thin-walled cystic mass with multiple calcifications may be observed, often filled with gelatinous mucinous material^[8]^; Ultrasonographic findings in female patients with LAMN typically present in 2 patterns: an oval, unilocular cystic mass in the right lower quadrant, exhibiting “jelly-like” and “onion skin-like” features, with partial visualization of the right ovary; a multilocular, irregular cystic mass in the pelvic or abdominal region, with a “jelly-like” consistency and poor delineation of the right ovary.^[9]^ These features may be easily confused with ovarian mucinous tumors or hydrosalpinx in female patients; thus, careful differential diagnosis is required. Additional diagnostic tools include endoscopy and magnetic resonance imaging (MRI). On endoscopy, only an external pressure indentation may be observed at the appendiceal orifice. In benign appendiceal mucinous tumors, MRI typically reveals a spherical lesion with well-defined margins; the cyst wall shows uniform enhancement, while the cystic fluid exhibits minimal or no enhancement.^[10]^

However, given the patient’s pregnancy, certain diagnostic procedures pose potential risks to the fetus. Abdominal CT and contrast-enhanced MRI are generally avoided due to the potential harm of ionizing radiation and the uncertain effects of gadolinium-based agents crossing the placenta. Therefore, preoperative evaluation primarily relied on abdominal ultrasonography, which is safe, noninvasive, and effective for assessing appendiceal pathology during pregnancy. Notably, the “onion skin sign” on ultrasound is regarded as a specific sonographic feature for diagnosing appendiceal mucocele.^[10]^ In the present case, prenatal ultrasonography at 30 weeks of gestation revealed a 76 × 21 × 26 mm anechoic cystic mass in the right lower quadrant, demonstrating concentric internal layering consistent with the “onion skin sign,” localized to the appendiceal region. These findings were consistent with the typical ultrasonographic features of LAMN. Furthermore, serial follow-up ultrasonography was performed throughout the remainder of the pregnancy, during which the size of the appendiceal mass remained stable with no evidence of rapid progression.

Surgical resection is currently considered the first-line treatment for LAMN, with simple appendectomy recommended for patients without extra-appendiceal involvement.^[11,12]^ In patients with rapidly progressing disease, pregnancy termination followed by definitive treatment may be required.^[13]^ In the present case, the patient developed abdominal pain and diarrhea at 27 weeks of gestation. At that time, symptoms were mild, inflammatory markers were within normal limits, electrolytes were normal, and tumor markers showed no significant abnormalities. The symptoms improved significantly with oral medication, supporting the decision to pursue expectant management. The pregnancy was subsequently monitored with regular follow-up until 40 weeks of gestation, during which no digestive symptoms recurred. Therefore, immediate surgical intervention at 27 weeks was deemed unnecessary.

Therefore, the decision to delay definitive surgical intervention until full-term delivery (40 weeks) was based on balancing fetal maturity with maternal safety, given that LAMNs typically progress slowly and carry a favorable prognosis. In asymptomatic patients without evidence of tumor growth or dissemination, expectant management is considered safe during pregnancy. Moreover, surgical intervention during the third trimester is associated with increased maternal and fetal risks. Coordinating surgical intervention with cesarean delivery minimized operative risks and enabled definitive treatment without compromising fetal outcomes.^[14,15]^

Given that the patient had reached full-term gestation, and in light of both the LAMN diagnosis and the high value placed on the pregnancy, the threshold for cesarean section was appropriately lowered to facilitate concurrent appendectomy. Preoperatively, tumor markers should be reevaluated for abnormalities, adequate fasting time ensured, and thorough bowel preparation completed. Intraoperatively, the LAMN should be isolated in situ, with gauze placed around it to prevent dissemination of mucinous epithelial cells. Extreme caution should be exercised during the procedure, with careful inspection for any residual mucin within the abdominal cavity. Bilateral adnexal structures should be routinely examined during the procedure. To minimize the risk of mucinous dissemination in suspected LAMN during pregnancy, we recommend: re-prepping/re-draping and using separate obstetric and general surgery instrument sets; routine wound protection and protective retrieval bags for specimen extraction; a no-touch technique (handle by mesoappendix, avoid grasping the cystic wall) with continuous low-pressure suction and field isolation by moist gauze; en-bloc resection to achieve negative margins without opening the lumen when the base is involved; copious irrigation with complete aspiration and systematic peritoneal survey; and glove/instrument changes before closure. Intraoperatively, macroscopic examination revealed a cystically dilated appendix measuring ~8 × 4 cm, with mucin accumulation within the lumen. The serosal surface appeared smooth, without ulceration, and no visible extraluminal mucin was observed. Histopathological evaluation confirmed features consistent with the 2012 diagnostic criteria established by the Peritoneal Surface Oncology Group International.^[16]^ LAMN is defined as a mucinous neoplasm with low-grade cytology and any of several distinguishing features. In the present case, the lesion exhibited a “pushing” pattern of invasion into the appendiceal wall, with acellular mucin extending in an expansile or diverticular fashion. A previously proposed classification system divides LAMN into type I and type II subtypes, with the present case corresponding to type I.^[17]^ Furthermore, a population-based study from the Netherlands on benign appendiceal mucinous neoplasm reported that the median interval from LAMN to PMP diagnosis is ~2 years. Notably, in 10% of patients, the interval exceeded 5 years, with the longest reported interval being 22 years.^[18]^ These findings underscore the need for long-term surveillance even in patients with histologically benign LAMN. Follow-up assessments should be conducted every 3 months during the first 5 years. After 5 years, the frequency of surveillance may be reduced, but follow-up should not be discontinued. During surveillance, both colonoscopy and full abdominal CT are recommended. In addition to imaging and histopathology, baseline serum levels of CEA, CA19-9, and CA125 offer valuable diagnostic and prognostic insights in appendiceal mucinous neoplasms. Elevated levels of any of these markers are consistently associated with poorer overall and progression-free survival. For example, in a retrospective cohort study involving 1338 patients with appendiceal adenocarcinoma, elevated preoperative levels of CEA, CA19-9, or CA125 were associated with significantly reduced 5‑year survival rates (82% vs 95%, 84% vs 92%, and 69% vs 93%, respectively, for elevated vs normal markers).^[19]^ Furthermore, in patients with PMP secondary to LAMN, elevated CA19-9 and CA125 levels have been associated with shorter progression-free survival.^[20]^ In the present case, all 3 tumor markers remained within normal limits preoperatively, consistent with a favorable prognosis. Immunohistochemical analysis further supported the diagnosis: the tumor cells were positive for CK19, CK20, and CEA, and negative for CK7, indicating a gastrointestinal mucinous origin. The Ki‑67 index (~20%) was low to moderate, consistent with low-grade proliferative activity. Notably, p53 expression was strongly positive (~80%), a feature uncommon in classic LAMN but possibly indicative of early molecular alteration rather than true high-grade transformation. Expression of mismatch repair proteins (MLH1, PMS2, MSH2, and MSH6) was preserved, indicating microsatellite stability and a low likelihood of Lynch syndrome.^[21]^

This case is notable for the prenatal diagnosis of LAMN at 27 weeks of gestation, based solely on ultrasonographic findings, followed by expectant management until term and definitive surgical intervention at 40 weeks. In contrast, most previously reported cases were diagnosed incidentally, either during cesarean section or during emergency laparotomy performed for acute abdominal symptoms in pregnancy.^[14]^ Moreover, in previously reported cases, management frequently involved early induction of labor or urgent surgery. In contrast, in the present case, close serial ultrasonographic monitoring enabled surgical deferral, thereby minimizing fetal risk without compromising maternal prognosis. Although no peritoneal dissemination was observed intraoperatively and final pathology confirmed low-grade LAMN, long-term surveillance remains essential. Published reports indicate that PMP can develop years after initial surgical treatment for LAMN, with reported intervals ranging from 1 to 22 years.^[16]^ Therefore, in accordance with Peritoneal Surface Oncology Group International principles, we propose a margin-/risk-stratified follow-up plan: For low-risk patients (R0 resection, no perforation, no extra-appendiceal mucin), perform history/physical and tumor markers (CEA, CA19-9, CA125) every 6 months for 5 years; obtain cross-sectional imaging (contrast-enhanced CT or MRI) annually for 5 years; and complete a baseline colonoscopy (if not recently performed), thereafter following population CRC screening recommendations. In the present case, surveillance at 2 weeks, 6 months, and 1 year postpartum was negative; we will maintain annual imaging through postoperative year 5 with semiannual tumor markers. If any high-risk features are present (positive/indeterminate margin, serosal acellular mucin, perforation, or intraoperative spillage), escalate surveillance to history/physical and markers every 3 to 6 months for the first 2 years, then every 6 to 12 months to year 5, with imaging every 6 to 12 months for 5 years (prefer every 6 months in years 0–2). If suspicious peritoneal disease is detected, refer to a peritoneal malignancy center, compute the PCI, and consider evaluation for CRS-HIPEC.

A review of this case highlights the clinical complexity of appendiceal mucinous neoplasms during pregnancy and the associated diagnostic and therapeutic challenges. On one hand, physiological changes during pregnancy, such as abdominal distension and discomfort, an mimic the symptoms of appendiceal mucinous neoplasms, leading to misdiagnosis or delayed diagnosis. On the other hand, the limitations of radiologic imaging and pharmacologic interventions during pregnancy further complicate both diagnosis and treatment. Therefore, for rare cases of pregnancy complicated by LAMN, MDT collaboration during prenatal care is of great importance for ensuring maternal and fetal safety, as well as for integrating multimodal diagnostic evidence. From an obstetric perspective, the integration of an MDT model into prenatal care allows for optimal utilization of cross-disciplinary expertise and is particularly suited to managing rare neoplastic conditions with complex prognoses. Due to the rarity of pregnancy complicated by LAMN, high-level clinical evidence remains limited. By integrating clinical observations, literature review, and retrospective analyses, sequential development of early diagnostic indicators can improve the quality of clinical reporting and help minimize bias, ultimately contributing to the establishment of reliable reference standards for early detection of LAMN during pregnancy.

## 4. Conclusion

This report presents a rare case of pregnancy complicated by LAMN. Given the patient’s pregnancy and the highly variable clinical presentation of LAMN, the risk of misdiagnosis underscores both the necessity and the challenges of differential diagnosis in such cases. Characteristic ultrasonographic features are of particular diagnostic value. Intraoperatively, the key concern is to prevent dissemination of mucoepithelial cells. Histopathological examination remains the gold standard for diagnosing LAMN. With multidisciplinary management during pregnancy and a coordinated surgical approach involving obstetric and gynecologic surgeons, the patient’s tumor remained stable without rupture or progression to PMP, and a healthy infant was successfully delivered via cesarean section at 40 weeks of gestation. To optimize maternal and fetal outcomes, further research is needed to improve understanding of this rare condition, enabling obstetricians and gynecologists to be better prepared for potential complications.

## Author contributions

**Data curation:** Beijiao Wu.

**Formal analysis:** Beijiao Wu.

**Methodology:** Beijiao Wu.

**Project administration:** Beijiao Wu.

**Writing – original draft:** Beijiao Wu.

**Writing – review & editing:** Beijiao Wu, Ling Ai.
